# Phosphated Cellulose as an Efficient Biomaterial for Aqueous Drug Ranitidine Removal

**DOI:** 10.3390/ma7127907

**Published:** 2014-12-09

**Authors:** Roosevelt D. S. Bezerra, Márcia M. F. Silva, Alan I. S. Morais, Josy A. Osajima, Maria R. M. C. Santos, Claudio Airoldi, Edson C. Silva Filho

**Affiliations:** 1 Federal Institute of Education, Science and Technology of Piauí (IFPI), Campus Teresina-Central, 64000-040 Teresina, Piauí, Brazil; E-Mail: rooseveltdsb@ifpi.edu.br; 2 Interdisciplinary Laboratory for Advanced Materials, Federal University of Piauí (UFPI), 64049-550 Teresina, Piauí, Brazil; E-Mails: marciaf3004@yahoo.com.br (M.M.F.S.); bredyalan@gmail.com (A.I.S.M.); josyosajima@ufpi.edu.br (J.A.O.); mrita@ufpi.edu.br (M.R.M.C.S.); 3 Institute of Chemistry, University of Campinas, Unicamp, P.O. Box 6154, 13083-971 Campinas, São Paulo, Brazil; E-Mail: airoldi@iqm.unicamp.br

**Keywords:** trimetaphosphate, cellulose, phosphatation, sorption, ranitidine

## Abstract

Crystalline cellulose chemically modified through a reaction with sodium trimetaphosphate (STMP) in an acidic or basic condition yielded Cel-P4 and Cel-P10. These phosphated solids were characterized by elemental analysis, X-ray diffraction (XRD), infrared (IR) spectroscopy, scanning electron microscopy (SEM), nuclear magnetic resonance (NMR) at the solid state for phosphorus nucleus and dispersive X-ray energy. The elemental results demonstrated that the phosphorylation reaction was more efficient in the basic medium, as supported by the amount of phosphorous content. The synthesized biomaterials decreased in crystallinity in comparison to the precursor cellulose, with an increase in roughness and present two distinct phosphorus environments in the formed structure. The phosphated cellulose in an alkaline condition was applied to sorb the drug ranitidine. This process was applied in varying pH, time, temperature and concentration. The best sorption kinetic model to fit the experimental data was the pseudo-second-order with a coefficient correlation of 0.8976, and the Langmuir isotherm model was the most adjusted to the variation in concentration. The efficient drug sorption has a low dependence on temperature, with maximum values of 85.0, 82.0 mg and 85.7 mg·g^−1^ for Cel-P10 at 298, 308 and 318 K, respectively. The best sorption occurred at pH = 6 with a saturation time of 210 min.

## 1. Introduction

**Figure 1:**
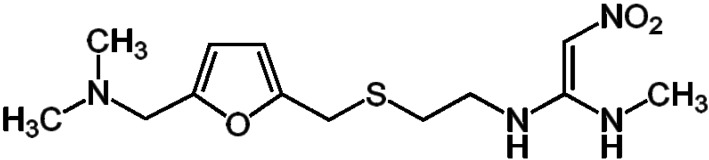
Molecular structure for ranitidine.

Ranitidine (Figure 1) belongs to a group of drugs formed by histamine-2 blockers, which reduce the amount of acid in the stomach. However, it is classified as a high environmental concern due to its occurrence in a variety of aquatic systems. For example, recent investigations identified it in aquatic environments, such as in water treatment plants, with a mean concentration that reached 288.2 ng·L^−1^ [1].

Under ideal conditions some investigations demonstrated that this drug is partially biodegradable with an efficiency of 71% of decomposition within 28 days. Consequently, the partial degradation over time prevents the elimination of the drug in water treatment plants whose purpose is to immediately complete removal to avoid accumulation in the surface waters. When present, sunlight can change the original structure of the drug, which generates persistent and toxic photoproducts [1].

There are some significant investigations associated with water disinfection through chloramination in the presence of aquatic ranitidine. The environments can be contaminated by nitrosamine precursor derives that are well-known as carcinogenic compounds. In addition, ranitidine showed strong potential to form *N*-nitrosodimethylamine (NDMA) which is considered a toxic and carcinogenic compound [2], with a molar yield superior to 77% of NDMA, through this drug varied from 0.10 g·L^−1^ to 15.7 g·L^−1^. Therefore, ranitidine together with twelve drugs at low levels of concentration (ng·L^−1^) caused growth inhibition of human embryonic HEK 293 cells with a decrease of 30% in cell proliferation as compared to controls [3,4].

Due to the presence of a variety of drugs in the environment, numerous techniques have been developed in order to remove these contaminants from wastewaters; however, many of them are not biodegradable and therefore, are not efficiently removed by traditional methodologies [5]. Amongst innumerable techniques, the sorption process has been widely used in effluent treatments for textile industries [6], leather industries [7], landfills [8], and other industries. However, only few investigations are associated with sorption, and the corresponding results related to such kinds of equilibrium in aqueous solutions.

As it is known, sorption is a physicochemical procedure in which components of gas or liquid phases are transferred or sorbed onto the sorbent solid surface to perform either as physical or chemical sorptions characterized the physisorption and chemisorption procedures. Various natural or synthesized materials are normally used as sorbents, such as activated carbon, chitosan, zeolites, clay, cellulose, ash, and natural oxides, among others [9].

Natural polysaccharides such as cellulose and chitosan have been used in various fields to create new materials due to their advantageous properties mainly the nontoxicity, biocompatibility, and biodegradability [10,11]. Cellulose, when conveniently modified through a series of reactions, has been widely used as a substrate for appropriate reagent immobilizations, which has numerous applications such as ion sorption, supports for microorganism immobilizations, removal of organic pollutants, sorption of surfactant molecules, and several other applications [12]. In this context, when cellulose has been chemically modified by incorporating various active groups, the new biopolymers have been widely explored because these centers, among other properties, increased the sorption capacity of the pulp, providing good sorption to proteins and metals [10,13,14].

The progress in research related to cellulose derivatives enabled the acquisition of new biopolymers such as those containing phosphate, which functional moiety evokes substantial research attentions. The chemical incorporation of phosphate into the cellulose structure significantly alters its property to give to the synthesized material characteristics associated with the immobilized group. The importance of this kind of biopolymer containing phosphate was developed to make textiles and cellulose-based flame retardants. From the chemical point of view, it has also been used as a material for cation exchange treatment of calcium-related diseases. Due to the capacity of cellulose phosphate to induce the calcium phosphate formation, it has been utilized as a biomaterial for possible biomedical applications and the inorganic functionality is very useful to connect multiple biologically active species to obtain useful surfaces [15]. Relevant applications concerned with phosphated cellulose are related in the form of membranes to sorb ovarian cells rather than the pure cellulose [16]. The increase in functionality was explored to transition metal removal [10], such as Fe^3+^, Cu^2+^, Mn^2+^, Zn^2+^, and Co^2+^, followed by La^3+^. However, its capacity in macromolecule removal like lysozyme, mioglobin, hemoglobin and albumin was also elucidated [13].

The present investigation aims to the synthesize cellulose phosphate by using trimetaphosphate with a variation in pH. The products of the synthesis were characterized and applied in order to study sorption of ranitidine by varying pH, time, temperature, and concentration. The data were adjusted to established models and also to different physicochemical kinetics applied to the obtained isotherms.

## 2. Results and Discussion

The low reactivity of natural cellulose was explored in specific reactions with its pulp (Cel-OH) to introduce phosphate groups in the polymeric structure. The success of this reaction consists in using the potentiality of phosphorus pentoxide (P_2_O_5_), triethylphosfate ((CH_3_CH_2_)_3_PO_4_) and phosphoric acid (H_3_PO_4_) reagents in hexanol as a solvent. However, another widely used process considered the reaction of cellulose with H_3_PO_4_ in *N*,*N*-dimethylformamide [10,13,14], as generally expressed by the reaction:

(1) $$\text{Cel-OH}+{\text{H}}_{3}\text{P}{\text{O}}_{4}\to \text{Cel-P}(\text{O}){\left(\text{OH}\right)}_{2}+{\text{H}}_{2}\text{O}$$

### 2.1. Characterization

Similar infrared (IR) spectra of pure cellulose and the chemically modified products were named Cel-P4 and Cel-P10. The precursor biopolymer presented a set of well-established bands; with the broadest one at 3437 cm^−1^ which is attributed to OH stretching vibration. The CH stretching vibration is assigned at 2899 cm^−1^, while the vibration at 1642 cm^−1^ referred to angular deformation of primary and secondary OH groups attached to the polysaccharide structure. The bands located at 1162, 1115 and 1061 cm^−1^ corresponding to the C–O stretching and those at 1435, 1370 and 1325 cm^−1^ are assigned to the presence of CH_2_ group. Those bands below 1000 cm^−1^ are attributed to the alcoholic absorptions; however, both biopolymers chemically showed the appearance of a shoulder of low intensity at 1240 cm^−1^ that corresponds to P=O stretching. The shift of the band at 1027 cm^−1^ of pure cellulose to 1034 cm^−1^ in case of Cel-P4 and 1036 cm^−1^ for Cel-P10, with the increased in intensity, refers to P–O–C deformation. The increase intensity in the 1125–985 cm^−1^ region in the spectra for both chemically modified celluloses is a clear indication of the presence of phosphate group in the new synthesized biopolymers. The IR spectroscopy is not an equivocal technique for this investigation to conclude about the chemical modification of cellulose structure since the typical bands of phosphates are usually most pronounced in the area of the spectrum where the pulp already has several intense bands in the 900–1200 cm^−1^ interval [17,18,19,20,21].

**Figure 2:**
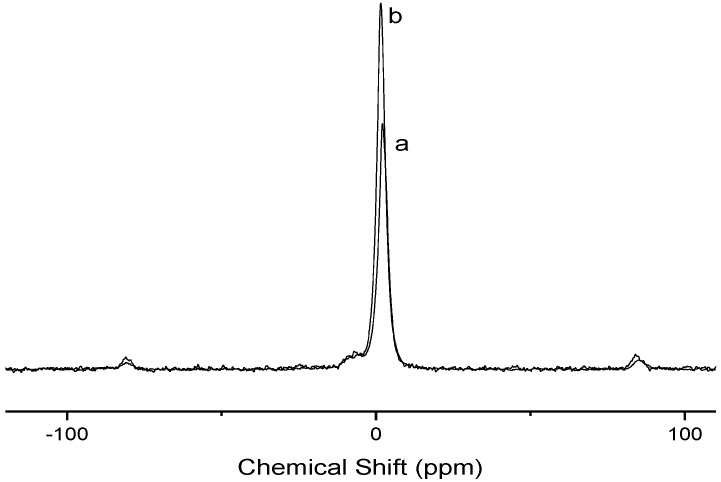
^31^P nuclear magnetic resonance (NMR) spectra of (**a**) Cel-P10 and (**b**) Cel-P4.

Magnetic resonance in the solid state for the phosphorous nucleus revealed the presence of two signals associated with this atom bonded to cellulose at 2.05 and −7.96, and at 1.45 ppm and −6.79 ppm spectra for Cel-P10 and Cel-P4 biopolymers, respectively, as shown in Figure 2, whose signals confirm the success of the phosphorylation reaction. These signals can be related to two different bonds represented by Cel-OPO-Cel and Cel-OP-ONa for Cel-P10, and to Cel-P-Cel-OH and P-ONa in Cel-P4. It has been observed that the intensity of the main peak for both biomaterials is distinct which could be due to analysis time of each material in the instrument. Both symmetrical sidebands approximately at 80 ppm and −80 ppm resulted from the rotational anisotropy power [15,17,22].

**Figure 3:**
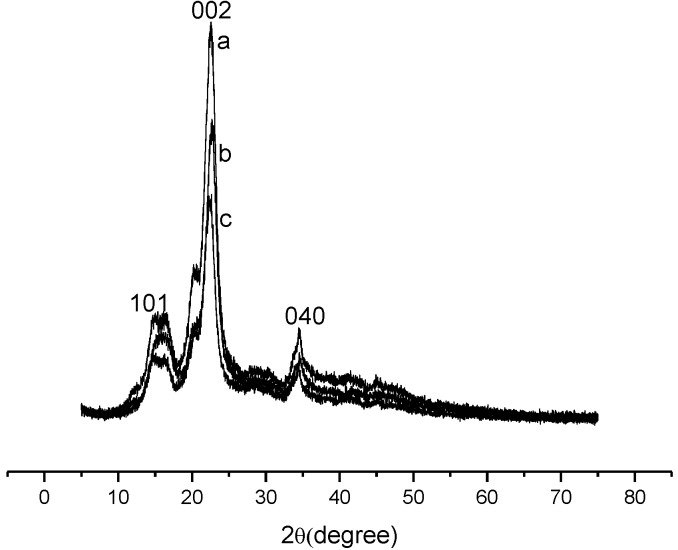
X-ray diffraction (XRD) patterns of (**a**) pure cellulose; (**b**) Cel-P10; and (**c**) Cel-P4.

X-ray diffraction (XRD) data demonstrated that the crystallinity of pure cellulose changes after phosphorilization due to the change of precursor biomaterial structure, as shown in Figure 3. For precursor cellulose a set of three crystallographic planes (101), (002), and (040) was observed which is a distinct characteristic for microcrystalline cellulose [23], as shown in Figure 3a. After reaction, the crystallinity of the chemically modified celluloses decreased as a function of the change in the number of intramolecular and intermolecular hydrogen bonds [24,25,26]. Possible crosslinking could further affect the interaction of the cellulosic structure.

**Figure 4:**
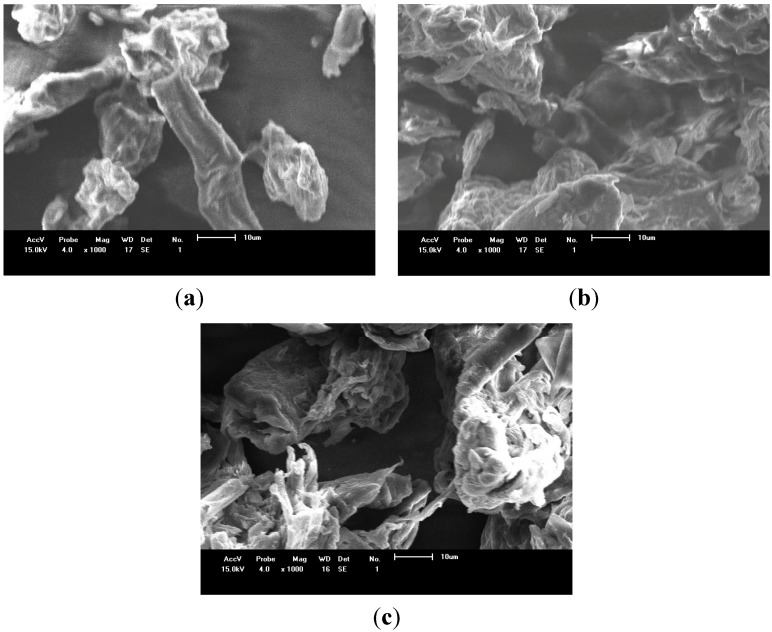
Scanning electron microscopy (SEM) of (**a**) pure cellulose; (**b**) Cel-P10; and (**c**) Cel-P4.

The surface morphology of the biopolymers can be elucidated from the scanning electron microscopy (SEM) images, as shown in Figure 4. As observed, both chemically biomaterials had their surfaces changed when compared with the pure cellulose, with an increase of the roughness, being more pronounced for Cel-P10.

The amount of phosphorus atoms immobilized in these biopolymeric structures was determined by X-ray energy dispersive spectroscopy, as listed in Table 1, which illustrates the results that confirmed the presence not only of phosphorus, but also of sodium in the two modified surfaces. In addition, there was also an increase of oxygen percentage, indicating the replacement of the original OH group in the cellulose by phosphate group [24], whose incorporation is directly dependent on pH. Thus, the increase in pH favors phosphate group incorporation as was previously observed for chemical modification for starch, which has a similar structure [27].

**Table 1:** Percentages of carbon, oxygen, sodium and phosphorous for pure cellulose, Cel-P4 and Cel-P10.

Element	Pure cellulose	Cel-P-4	Cel-P-10
C	72.1	63.9	60.8
O	27.9	35.7	36.4
Na	-	0.2	1.6
P	-	0.2	1.2

**Figure 5:**
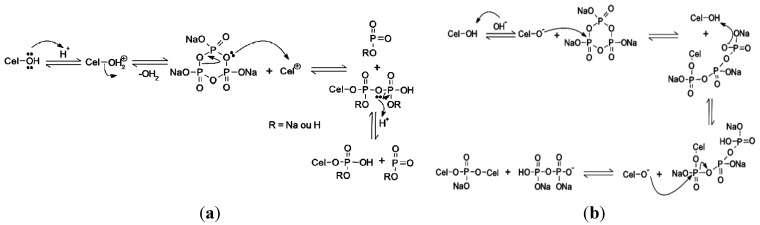
Proposed mechanisms of the reaction of cellulose with sodium trimetaphosphate (STMP) in the (**a**) acidic and (**b**) basic conditions.

The lowest phosphorus ratio found for Cel-P4 can be associated with the reaction mechanism that could occur in both processes. In the acidic condition, the proton can easily bond to the active Cel-OH site on cellulose to form Cel-OH_2_^+^, whose OH_2_^+^ group can be released to be a better leaving group than OH, thus forming Cel^+^. It is easily attacked by electron oxygen pairs of sodium trimetaphosphate (STMP) to form a covalent bond, as illustrated in Figure 5a. When in the basic medium, Cel-OH loses hydrogen that can bond OH^−^ species, and consequently, Cel-O^−^ attacks the metaphosphate positive site which then forms a covalent bond between the phosphorus and the cellulosic structure, as shown in Figure 5b. Thus, these different mechanisms govern the quantity and types of phosphates formed after the phosphorylation reaction [27].

The results listed in Table 1 showed that the percentage of the incorporated phosphorus was practically the same as sodium, indicating that the majority of the sodium present in the modified biomaterials from the metaphosphate reagent was used in the reaction. The results were obtained from energy dispersive X-ray spectroscopy (EDS)/SEM, which were selected to quantify only carbon, phosphorus, sodium and oxygen elements.

For the chemically modified biomaterial Cel-P4 in the acidic medium, the percentage of sodium is slightly less than the amount of phosphorus, indicating that during the reaction, sodium presented in metaphosphate can be replaced by a proton from the solution, as shown in Figure 5a. On the contrary, in the basic medium, the amount of sodium is higher than phosphorus, which indicates that it could be generated from sodium hydroxide used to adjust the pH of the medium to reach pH = 10.

### 2.2. Ion Exchange

The capacity of ionic exchange for the three adsorbents (pure cellulose, Cel-P4, and Cel-P10) are shown in Table 2. The ionic exchange capacity for the three materials was similar, showing that there is some difference but only a small variation among the materials due to the number of incorporated phosphate groups shown in Table 1. Figure 5 demonstrates the way they were incorporated.

**Table 2:** Ion Exchange capacity of each adsorbent in the sorption test.

Adsorbents	Ion exchange capacities (meq·g^−1^)	Deviation
Pure cellulose	0.0119	0.0002
Cel-P4	0.0121	0.0007
Cel-P10	0.0124	0.0002

### 2.3. Sorption

#### 2.3.1. Influence of pH

**Figure 6:**
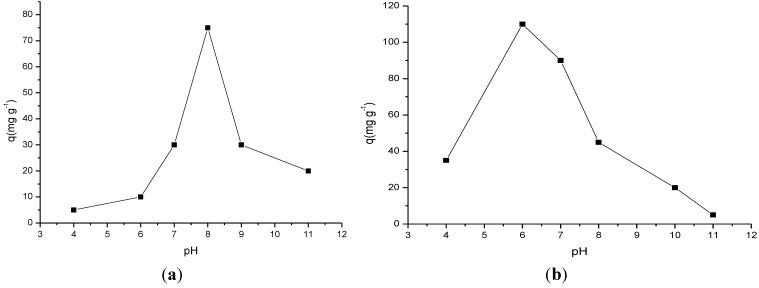
Influence of pH in ranitidine sorptions on (**a**) Cel-P4 and (**b**) Cel-P10.

Both chemically modified cellulose biopolymers were applied to ranitidine sorption with a variation in pH in order to determine the best sorption and based on this result to choose the highest surface capacity to use in this process. Thus, for Cel-P4 it was observed that the sorption increased to reach pH = 8, as shown in Figure 6a. This behavior may have occurred because the drug in an aqueous solution acquires a partly positive charge due to the easy basic nitrogen center in the drug structure. As expected, in the acidic medium the excess of available protonated basic centers can electrostatically favor the interactions with the active phosphated cellulose sites rather than the drug, to form uncharged cellulose phosphate Cel-P-OH and, consequently, disfavor the sorption. Thus, as the pH increases the amount of protons decreases, causing an enhancement in sorption due to the appearance of free phosphonate anion that can electrostatically interact with the protonated basic amine centers to reach a maximum value and from this point, decrease in drug sorption for both sorbents. However, the decrease in sorption from the highest pH value expresses the facility of protons bonding to amino groups, which causes an abrupt decrease in value. This behavior reveals that in the basic condition the excess of hydroxyl anions has the ability to remove drug positive sites to cause a decrease in sorption. These results are in agreement that drug/phosphated cellulose interaction is electrostatic in nature, which involves the negative charge located in the phosphate group and the opposite charge sited on nitrogen group of the drug [10,14,28,29], whose investigation to explore the effectiveness of this drug depends on the pH medium.

Based on the results in Figure 6b, the variation in pH demonstrated that the sorption for Cel-P10 increased to the maximum value at pH = 6 and from there, decrease nearly linearly until pH = 11. The sorption drug mechanism for Cel-P10 is very similar to that one for Cel-P4. As illustrated, the highest sorptions in both cases occurred at near neutral pH, in which condition the phosphate groups of the chemically modified cellulose can easily be deprotonated. Consequently, the negative charges in phosphate groups can electrostatically interact with the opposite positive charge, mainly those related to the most basic centers that are easily protonated, as represented by drug nitrogen centers, to enhance the sorptions to give the maximum values at pH = 6.

The amount of drug sorbed on both chemically modified biopolymers occurred at pH = 8 and pH = 6 for Cel-P4 and Cel-P10, respectively. This behavior suggested that a minimum pH adjustment is required for the application of both biomaterials in wastewater treatment. These biomaterials could even be applied without pH adjustment depending on the natural condition of the effluent. This process would require only a minor expenditure for the implementation of this technique in water treatment plants. Taking into account the maximum sorbed values of 110 mg·g^−1^ and 75 mg·g^−1^ for Cel-P10 and Cel-P4, the comparison clearly suggested the sorption process depends on the quantity of phosphate groups attached to the original biopolymer, as was observed for highest value for the first sorbent. Another important feature to consider is the increased ratio of the percentages of oxygen and sodium atoms incorporated in the biopolymer. For example, Cel-P10 had higher percentages of both atoms that were reflected in the sorbed amount. As also observed, oxygen atoms are included in the precursor biopolymer after phosphorylation process. The excess of sodium was released during the washing procedure, but the large amount of oxygen is responsible for drug sorption.

For pure cellulose, the maximum interaction occurred at pH = 11 with a value sorbed of 36 mg·g^−1^ through electrostatic interactions [30].

These pH data were supported by the data presented in the ionic exchange. Even though there was an ionic exchange during the adsorption of the substance on the surface of the materials; it was not the predominant device, which means that the sites on which the ionic exchanges occurred were the same on the three adsorbents. This showed that the values of adsorption of the substance in pure cellulose, Cel-P4, and Cel-P10 occurred predominantly due to the interaction between the sites on which there was phosphatizing. Therefore, there was an increase of the negative charge on the cellulose, after phosphatizing, which helped the electrostatic interaction between the phosphatized cellulose and the substance [13,14].

#### 2.3.2. Influence of Time

**Figure 7:**
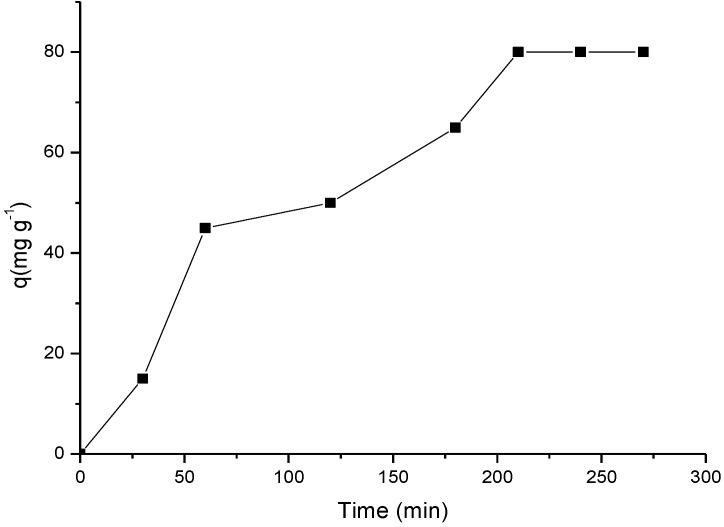
Sorption isotherm of ranitidine as a function of time on Cel-P10, in pH.

The kinetic study was applied to the chemically modified biomaterial Cel-P10 that had the best sorption at pH = 6 for the ranitidine 1000 mg·L^−1^ solution, and the required time for this system to reach the equilibrium saturation was 210 min, as shown in Figure 7.

#### 2.3.3. Influence of Temperature and Concentration

**Figure 8:**
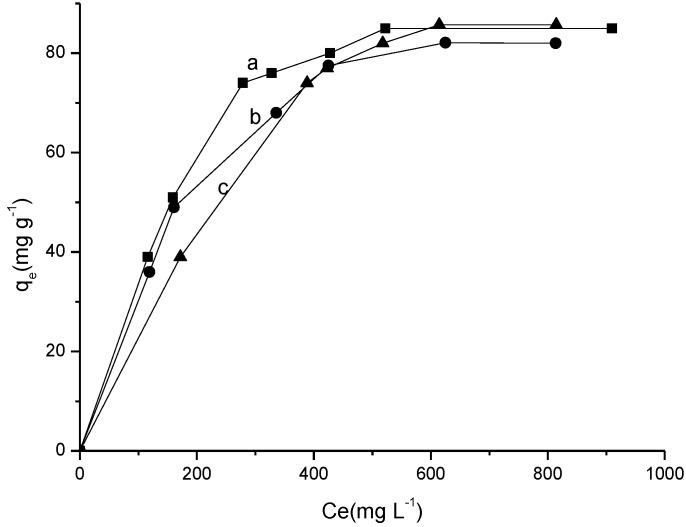
Sorption isotherms of ranitidine on Cel-P10 at (**a**) 298 K; (**b**) 308 K; and (**c**) 318 K; at 210 min and pH = 6.

The sorption isotherms were carried out by varying the temperature and solution concentration for Cel-P10 at room temperature and other temperatures to show the sorption behavior. The results are also included in Figure 8. These data were adjusted to the Langmuir, the Freundlich, and the Temkin models [31,32,33,34,35,36,37].

The isotherms that performed at 298 ± 1, 308 ± 1 and 318 ± 1 K gave the maximum quantity sorbed of 85.0, 82.0, and 85.7 mg·g^−1^, respectively. For the pure cellulose the maximum quantity sorbed of 28.1, 32.1, and 32.9 mg·g^−1^ for 298 ± 1, 308 ± 1 and 318 ± 1 K, respectively [30]. The calculated data from the linearized equations is listed in Table 3. These data enable the evaluation of the best experimental results to fit the sorption in order to find an appropriate model. Based on the correlation coefficient *R*^2^, it was observed that the Langmuir model gave the best fit. As established, this model for sorption occurred primarily in the saturated monolayer formation of solute molecules on the sorbent where the sorption affinity increases with sorbate concentration. This can be explained by the fact that the biomaterial surface is negatively charged which makes it easy to interact with the drug in an electrostatic interaction mechanism. Comparing the results for all three temperatures, it was observed that there was little variation in maximum sorption capacity, indicating that the temperature is not a variable that influences the sorption process too much [31,38]. With a maximum sorption capacity at 318 K, despite the small difference, the system is very well-adjusted to be used at room temperature. For this system *R*_L_ values are less than the unit for all temperatures that indicate the spontaneous drug/ranitidine sorption for Cel-P10. Although the Freundlich model does not have a good linear fit with the experimental isotherm, it can be said that from the same sorption the process is again spontaneous, as the *n*_f_ value is higher than the unit.

**Table 3:** Results for the isotherms of the Langmuir, the Freundlich and the Temkin related to ranitidine sorption on Cel-P10 in aqueous solution at 298 K, 308 K and 318 K.

Temperature (K)	Langmuir model				Freundlich model			Temkin model		
	*K*_L_	*q*_m_	*R*^2^	*R*_L_	*K*_F_	*n*_f_	*R*^2^	*K*_T_	*n*_t_	*R*^2^
298	0.243	125.0	0.9771	0.008	7.210	2.577	0.8270	6.18 × 10^−30^	0.042	0.8417
308	0.296	104.0	0.9871	0.005	5.272	2.342	0.9151	7.82 × 10^−35^	0.040	0.9375
318	0.183	125.0	0.9414	0.009	2.769	1.872	0.8814	5.69 × 10^−59^	0.031	0.8959

For sorption of ranitidine in pure cellulose, the best linear fit occurred for the Freundlich model because the type of interaction for this system was electrostatic [30].

The efficiency in drug removing is very favorable for chemically modified cellulose when compared with pure cellulose which presented a maximum value of only 32.9 mg·g^−1^ in pH = 11 at the highest temperature assayed, which gives an enormous increase near 260% after phosphorylation without considering the influence of temperature. Also, the variation in pH is so small when compared to pure cellulose [30].

#### 2.3.4. Biopolymer/Ranitidine Interaction

**Figure 9:**
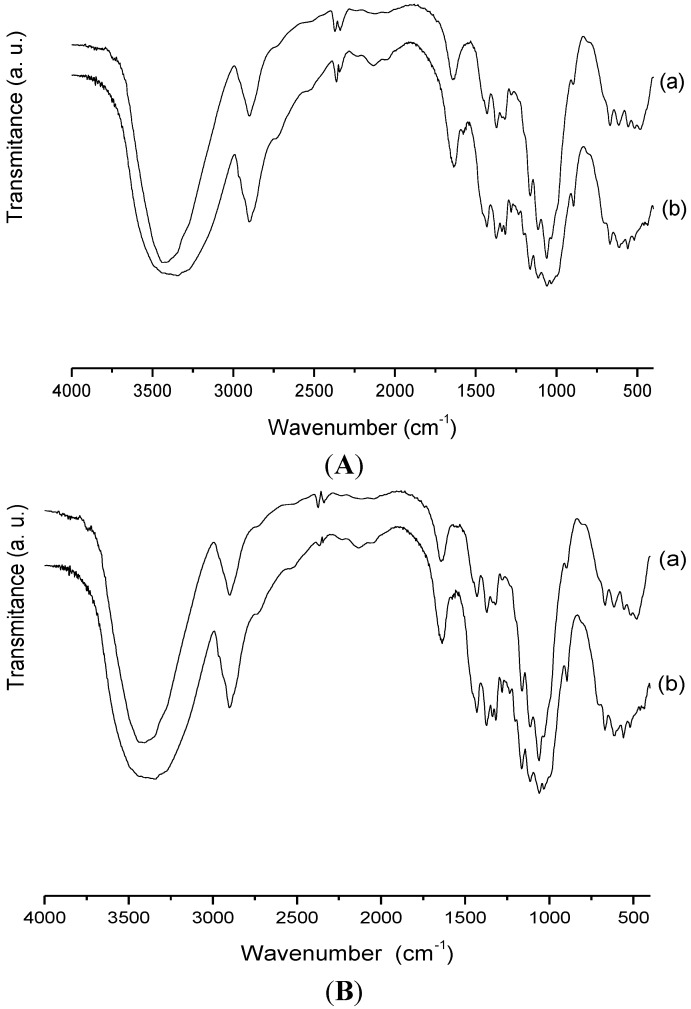
Infrared (IR) spectra of (**A**) CelP-4 and (**B**) Cel-P10 (**a**) before and (**b**) after sorption with ranitidine.

To ascertain whether there was indeed the interaction of the drug on the surface of modified cellulose after the sorption test in more favorable pH sorption, the solids were dried and analyzed by IR and XRD. The results are shown in Figure 9A for IR for Cel-P-4 before and after sorption. It was observed that after the sorption two medium bands appeared at 1336 cm^−1^ and 1577 cm^−1^ that correspond to symmetric and asymmetric stretching for NO_2_, respectively. In addition, the appearance of a band at 1206 cm^−1^ and another at 1232 cm^−1^ for CN stretching of secondary amines were also observed. There are also five bands at 2048; 2132; 2230; 2527 and 2745 cm^−1^ that are related to NH stretching of secondary and tertiary ammonium salts, whose bands are directly related to Cel-P-4 drug surface, which proves that it was sorbed. The spectrum for Cel-P-10 in Figure 9B shows the appearance of the same bands of Cel-P-4 that also confirmed the drug was sorbed onto the surface [20,21]. The same interactions were observed in the spectrum for pure cellulose/ranitidine [30].

**Figure 10:**
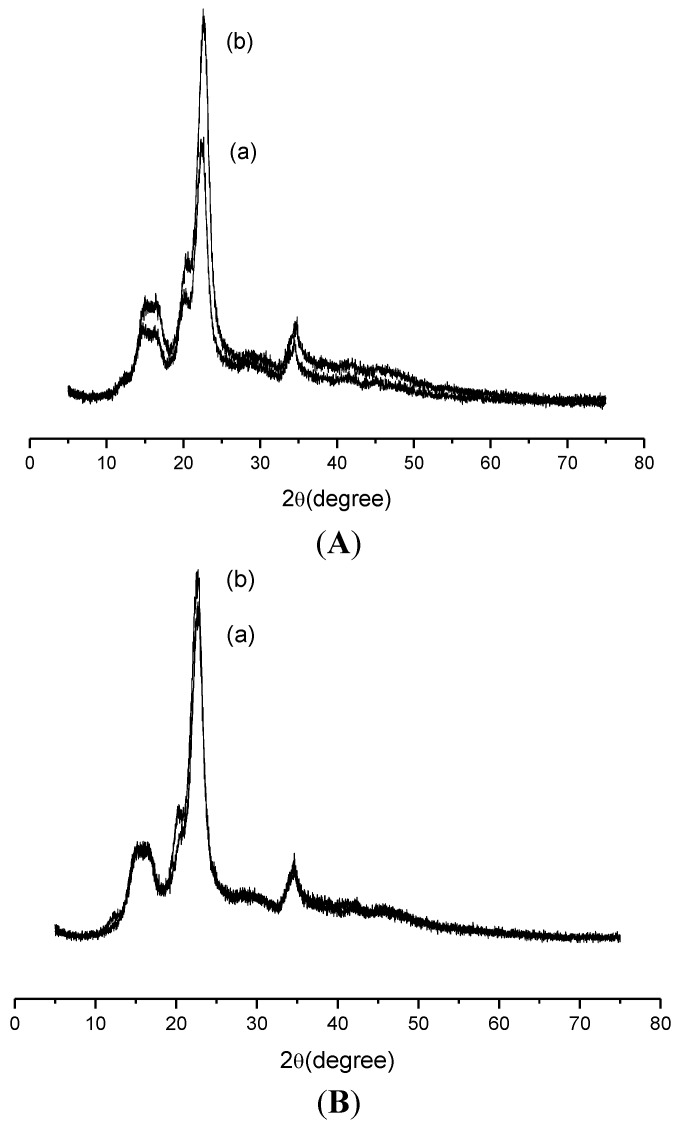
XRD of (**A**) CelP-4 and (**B**) Cel-P10 (**a**) before and (**b**) after sorption with ranitidine.

To analyze the crystallographic profiles after sorption, the Cel-P-4 and Cel-P-10 samples were submitted to XRD, as shown in Figure 10. It was observed that after variation in pH the expected profiles demonstrated an increase in crystallinity, indicating that their surfaces after sorption had a change that could be related to the fact that the drug is bonded to the material, although it was not as effective for the Cel-P-10. This higher increase in crystallinity for Cel-P-4 may be related to the presence of hydrogen in Cel-P-OH in the modified material structure, whose behavior does not happen in Cel-P-10 as given by Cel-P-ONa or Cel-P-O-P-Cel. The probable drug sorption through the active site of nitrogen increased the amount of intramolecular and intermolecular bonds. The same observations were observed in the XRD for pure cellulose/ranitidine [30].

## 3. Experimental Section

### 3.1. Reagents

Microcrystalline cellulose (Fagron, São Paulo, Brazil), STMP (Aldrich, São Paulo, Brazil), sodium sulfate (Synth, São Paulo, Brazil), sodium hydroxide (Synth), hydrochloric acid (Sigma, São Paulo, Brazil), ranitidine (Fagron, São Paulo, Brazil) were all of analytical grade and were used without prior purification. Deionized water was used as required.

### 3.2. Synthesis of Phosphated Cellulose

The microcrystalline cellulose used was previously dried at 373 K for 24 h in order to remove physically sorbed water [39]. After that, 6.8 g of STMP and 5.0 g of Na_2_SO_4_ were dissolved in 200 mL of water, and the pH was adjusted with 0.10 mol·L^−1^ of NaOH and 0.10 mol·L^−1^ of HCl, to give two solutions at pH = 4 and pH = 10. Shortly thereafter, 3.0 g of cellulose was added in these two different systems, the suspensions were stirred for 1 h at room temperature, and then placed in an oven for 6 h at 403 K. The withdrawn sample was placed in 100 mL of water and the pH was adjusted to 6.5, washed three times with water, vacuum filtered, and finally, was placed to dry in an oven at 333 K for 24 h [27,40]. The two biomaterials were termed Cel-P4 and Cel-P10, according to the pH of the synthesis.

### 3.3. Characterization

The IR spectra were obtained using Varian Model 660 IR Spectrometer (Madison, WI, USA) through tablet method using 1% of KBr in 32 scans, in the 4000–400 cm^−1^ region. Solid state spectra for ^31^P nuclear magnetic resonance (NMR) cross-polarization (CP) and magic angle spin (MAS) were obtained on a Bruker AC 300 to 121 MHz (Karlsruhe, Germany) using the following experimental conditions: acquisition time 45 ms, sequence pulse with contact time of 100 μs and pulse interval of 10 s. H_3_PO_4_ was used as a reference to calibrate the chemical shift scale. To verify microcrystalinity pulp used as well as their derivatives diffractograms were obtained using the Shimadzu Instrument, Model D600-XR A (Kyoto, Japan), 2θ in the range from 5° to 75°, scanning speed of 0.085°·s^−1^, using CuKα radiation source with wavelength 154.06 pm. The SEM was used to observe the surface morphology of pure and chemically modified cellulose using a JEOL Microscope, Model JSTM-300 (Peadbord, MA, USA). The elemental analysis were investigated using a SEM JEOL JSM 6360LV Apparatus, operating at 20 kV, coupled with an EDS. The phosphate cellulose were disperse in acetone solvent and deposited on a sample holder, allowing the solvent to evaporate, and by determining the percentage of C, Na, O and P present in the samples. The concentration of the drug and the spectra were obtained regarding this in a spectrophotometer ultraviolet-visible (UV-Vis) Cary Model 300, Varian (Palo Alto, CA, USA).

### 3.4. Ion Exchange Capacity

Adsorbent (0.1 g) was immersed in 50 mL of NaCl for 16 h through the ion exchange reaction, parts of H^+^ in the samples were substuted by Na^+^ and gave HCl solution. HCl solution was collected and titrated with a standard NaOH solution (5 mmol·L^−1^) [41].

### 3.5. Sorption

#### 3.5.1. Influence of pH

The influence of pH on drug sorption was conducted using 0.10 mol·L^−1^ of HCl and/or NaOH solutions to obtain ranitidine solutions with various pH values, whose concentration was near 1000 mg·L^−1^. For each determination, a volume of 20.0 mL of drug solution of known concentration was placed in contact with approximately 20 mg of the suspended sorbent at 298 K for 24 h. Then, the sorbent was separated from the solution by centrifugation of 3200 rpm for 15 min, and the concentration, determined by UV-Vis spectrophotometry, had a the wavelength of maximum absorption of the drug at 313 nm. The sorption capacity [20,42] was determined in each determination through Equation (2):

(2) $$q=\frac{V(C_{0}-C_{\text{f}})}{m}$$

where *V* (L) is the volume of drug solution, *C*_0_ (mg·mL^−1^) and *C*_f_ (mg·L^−1^) are the initial and final drug concentrations, and *m* (g) is the sorbent mass used.

#### 3.5.2. Influence of Time

The kinetics of drug ranitidine removal was performed in a batchwise process. Thus, aliquots of 20.0 mL of the original drug solution of 1000 mg L^−1^ in pH of higher sorption were suspended with approximately 20 mg of sorbent in 125 mL of erlenmeyer flasks. The suspensions stirred in a shaker at 298 K for several time intervals. After each determined time, the supernatant was separated by centrifugation as before [31,38,42,43,44] and identically determined through spectrophotometry whose amount of drug sorbed was calculated by Equation (2). The results obtained were adjusted to two kinetic models, pseudo-first order and pseudo-second order as shown in Equations (3) and (4):

(3) $$\text{log}(q_{\text{e,Exp}}-q_{t})=\text{log}q_{\text{e,Theo}}-\frac{{\text{k}}_{1}}{2303}t$$

(4) $$\frac{t}{q_{t}}=\frac{1}{h}+\frac{1}{q_{\text{e}}}t$$

where *q*_e_ (mg·g^−1^) is the amount sorbed for each gram of sorbent in the equilibrium, *q_t_* refers to amount sorbed in time *t* (min), and k_1_ (min^−1^) is a speed constant of sorption for pseudo-first-order. Plotting the graph of log(*q*_e,Exp_ − *q_t_*) *versus* time the values of equation of pseudo-first-order are obtained, *q*_e,Theo_ and k_1_ can be determined from the angular and linear coefficients, *h* (mg·g^−1^·min^−1^) value is the initial velocity of sorption when *t* → 0, as defined by:

(5) $$h=k_{2}{q_{e}}^{2}$$

From *t*/*q_t_*, as a function of *t* plot the linear and angular coefficients can be obtained to use in the *k*_2_ and *q*_e_ calculation values.

#### 3.5.3. Influence of Concentration and Temperature

The sorption isotherms were performed at 298 ± 1, 308 ± 1, and 318 ± 1 K, and the solution concentrations of the drug were prepared in the 100–1000 mg·L^−1^ range in the best pH sorption. Aliquots of 20.0 mL of each solution were suspended with about 20 mg of the sorbent as before. The suspensions were stirred at the corresponding temperature to obtain the respective isotherm. After completion, the supernatant was separated and the concentrations were determined as before and calculated through Equation (2). The experimental data were fitted to the Langmuir, the Freundlich and the Temkin models [31,32,33,34,35,36,37], and the linearized form of the Langmuir equation [31,33] is represented by Equation (6):

(6) $$\frac{C_{\text{e}}}{q_{\text{e}}}=\frac{1}{K_{\text{L}}q_{\text{m}}}+\frac{C_{\text{e}}}{q_{\text{m}}}$$

where *q*_e_ (mg·g^−1^) is the amount of solute sorbed per gram of sorbent, *C*_e_ is the concentration at solute equilibrium, *K*_L_ is the Langmuir constant that corresponds to the affinity between sorbent surface and solute, and *q*_m_ is a constant that represents the sorbate covering in a monolayer formation, to give the maximum observed solute.

If the system conforms to the Langmuir isotherm model, the *C*_e_/*q*_e_ as a function of *C*_e_ plot should produce a straight line, whose slope corresponds to 1/*q*_m_ and linear coefficient 1/(*K*_L_*q*_m_). The Langmuir data can be expressed in terms of a dimensionless separation factor, *R*_L_, defined by Equation (7), and thus can assess the shape of the isotherm:

(7) $$R_{\text{L}}=\frac{1}{1+\text{b}C_{\text{s}}}$$

where *C*_s_ is the highest drug concentration at equilibrium (mg·L^−1^), and b is the Langmuir constant. For a favorable sorption *R*_L_ values must lie between 0 and 1 (0 < *R*_L_ < 1), while *R*_L_ > 1 represents an unfavorable sorption. When *R*_L_ = 1 represents a linear sorption and for *R_L_* = 0, the sorption is irreversible [34].

The Freundlich model isotherm suggests that the sorption energy decreased logarithmically as the surface became covered by the solute, which differentiates it from the Langmuir model, as expressed by Equation (5) and the linear form by Equation (8):

(8) $$\text{log}q_{\text{e}}=\text{log}{\text{K}}_{\text{F}}+\frac{1}{n_{\text{F}}}\text{log}C_{\text{e}}$$

where K_F_ is a constant related to the sorption capacity, and *n*_F_ is also a constant related to the strength of sorption and spontaneity of the reaction. Values of *n* in the 1 < *n* < 10 range indicate favorable sorption, *C*_e_ and *q*_e_ has the same meaning of the Langmuir equation. The values of *n*_F_ and K_F_ may be obtained by log-linear graph of *C*_s_ as a function of *n*_F_, the slope of which is equal to 1/*n*_F_ and the linear coefficient is equal to K_F_ [35,38].

The Temkin model, which is a fairly simple model, considers that the heat of sorption decreases linearly with the coverage of the interaction of the sorbent and sorbate and through the linearized form as given by Equation (9), *q*_e_ value can be obtained:

(9) $$q_{\text{e}}=\frac{1}{n_{\text{T}}}\text{ln}{\text{K}}_{\text{T}}+\frac{1}{n_{\text{T}}}\text{ln}C_{\text{s}}$$

where *n*_T_ indicates quantitatively the reactivity of the energetic sites of the material, and K_T_ is a constant that includes the equilibrium constant. This model considers the system close to the one proposed by Langmuir [36,37].

## 4. Conclusions

In searching new materials that could be chemically modified, cellulose is appropriate when a desirable function is attached, as takes place with phosphate from the original STMP reagent. The highest amount of phosphorus percentage attached to the polysaccharide varied as a function of basic pH = 10, whose biomaterials obtained were efficient in sorbing the drug from aqueous solutions. Again, for this process the variable pH influences the ranitidine removal which presents the maximum values for chemically modified cellulose containing high phosphorous percentage at neutral pH and for the other sample with lower amount of phosphorus at pH = 8. The more efficient kinetic sorption for the drug/chemically modified cellulose with the first biopolymer was established the equilibrium at 210 min, whose data were adjusted to the pseudo-second order model. The sorption as a function of temperature demonstrates some independence on this variable and the data was mostly adjusted to the Langmuir model with a spontaneous process. The maximum sorption capacity of 85.7 mg·g^−1^ showed that the chemically modified cellulose increased the sorption drastically to reach nearly 260% in comparison with the pure cellulose, which suggests that these kinds of biopolymers are promising sorbents for removing the ranitidine pollutant from aquatic environments.
